# Warning message of trendless fatality rates (case study: Yazd rural roads)

**Published:** 2019-08

**Authors:** Abolfazl Khishdari

**Affiliations:** ^*a*^University of Yazd, Yazd, Iran.

## Abstract

**Background:**

Road traffic death have devastating impact on society. Decade of action has aimed to save many lives from this issue. Iran has established its own decade of action strategic plans that is to reduce yearly fatality rates by 10%. Despite being the heart of traveling of Iran from and to, Yazd Province is amongst those whom have experienced low fatality rates compared to other provinces of Iran. Fortunately, this year (2018) traffic fatality rate has reduced by 17% compared to last year. This article aimed to investigate the possibility of using ARIMA model to track monthly fatality rate based on 5 years of Yazd rural road hospital fatal accidents data.

**Methods:**

The ARIMA models were investigated using iteration procedure. ARIMA models components (i.e. AR, I and MA) varied in the range (0, 5). Their goodness of fits were investigated using akaike-information-criterion (AIC). Anaconda Jupyter-notebook used for ARIMA modeling.

**Results:**

Based on p-values of both ljungbox test and boxpierce test, and also autocorrelation function graph, it was found that there is non-autocorrelation between the series, in 95% confidence level indicating that there is no correlation between monthly fatal accident frequencies in province scale. Also insisted that data are stationary as first order time series differentiating totally diminished the autocorrelation function graph.

**Conclusions:**

Being stationary, fatality rates are more or less near the same in time series plots. This is a warning message to stakeholders to take in to account and use more elaborate and sophisticated transportation safety action plans to reduce road traffic fatalities in future. Using ITS related technologies might be the primary remedy to reduce road fatalities.

**Keywords:**

Fatalities, ARIMA, warning

